# The interventional direct peroral cholangioscopy toolbox for endoscopic snare resection of a high-grade biliary intraductal neoplasia

**DOI:** 10.1016/j.vgie.2022.10.007

**Published:** 2022-11-29

**Authors:** Jerry Yung-Lun Chin, Zongming Eric Chen, Mark D. Topazian, Andrew C. Storm

**Affiliations:** 1 Division of Gastroenterology and Hepatology, Mayo Clinic, Rochester, Minnesota; 2 Division of Anatomic Pathology, Mayo Clinic, Rochester, Minnesota; 3 Division of Gastroenterology and Hepatology, Mayo Clinic, Rochester, Minnesota

**Keywords:** CO_2_, carbon dioxide, DPOC, direct peroral cholangioscopy, RFA, radiofrequency ablation

## Abstract

Video 1 Demonstration of techniques for performing direct peroral cholangioscopy.

Demonstration of techniques for performing direct peroral cholangioscopy.

## Background and Aims

Direct peroral cholangioscopy (DPOC) is a valuable diagnostic and therapeutic tool for various biliary disorders because it allows direct endoscopic visualization of biliary lumen and mucosal abnormalities. Direct cholangioscopy carries several benefits, including high-definition imaging with the availability of narrow-band imaging, lower operating expenses and cost, and the ability to use a wide range of accessories through the working channel.^1^^,^^2^ In this article, we demonstrate endoscopic techniques for performing biliary intraductal polypectomy using the ultra-slim pediatric gastroscope (GIF-H190N; Olympus America, Center Valley, Pa) and showcase the various endoscopic accessories available for use.

## Endoscopic Techniques

**Figure 1:**
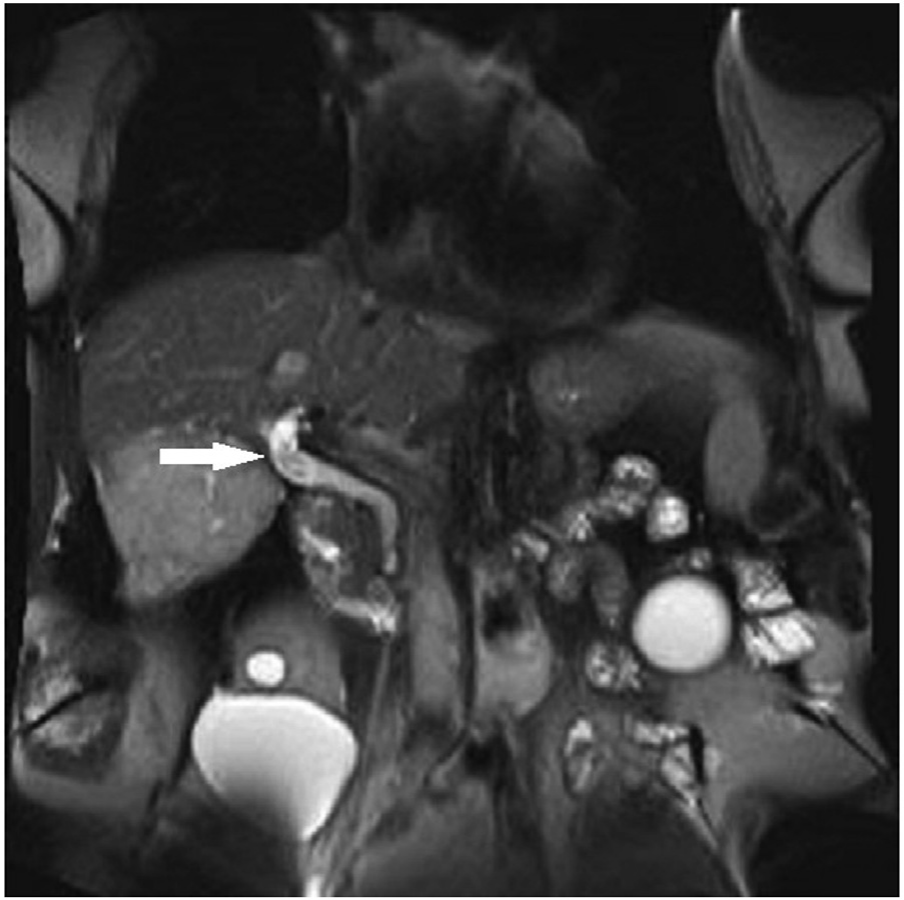
MRCP demonstrating multiple large filling defects within the biliary system (*white arrow*).

**Figure 2:**
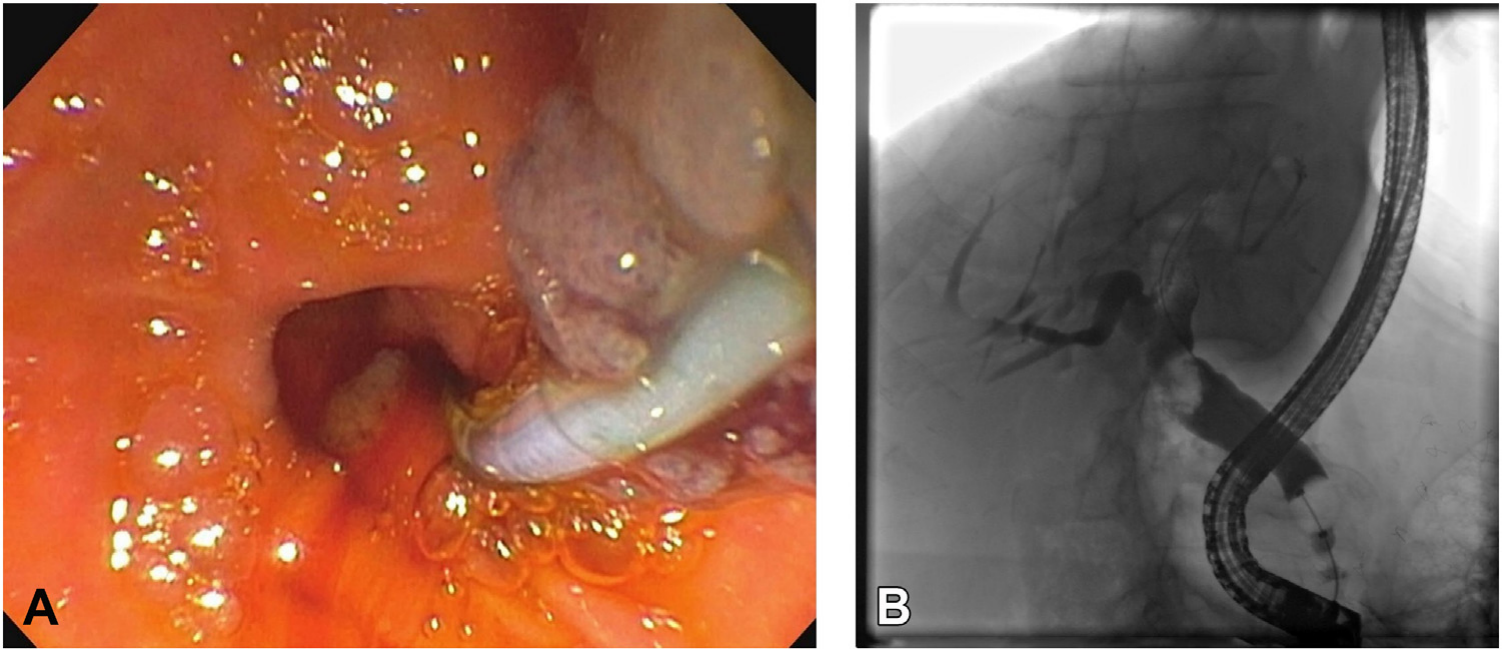
**A,** Polypoid material extracted from the biliary tree during index ERCP. **B,** Initial cholangiogram demonstrating large filling defect at the level of the common hepatic duct, just below the bifurcation.

**Figure 3:**
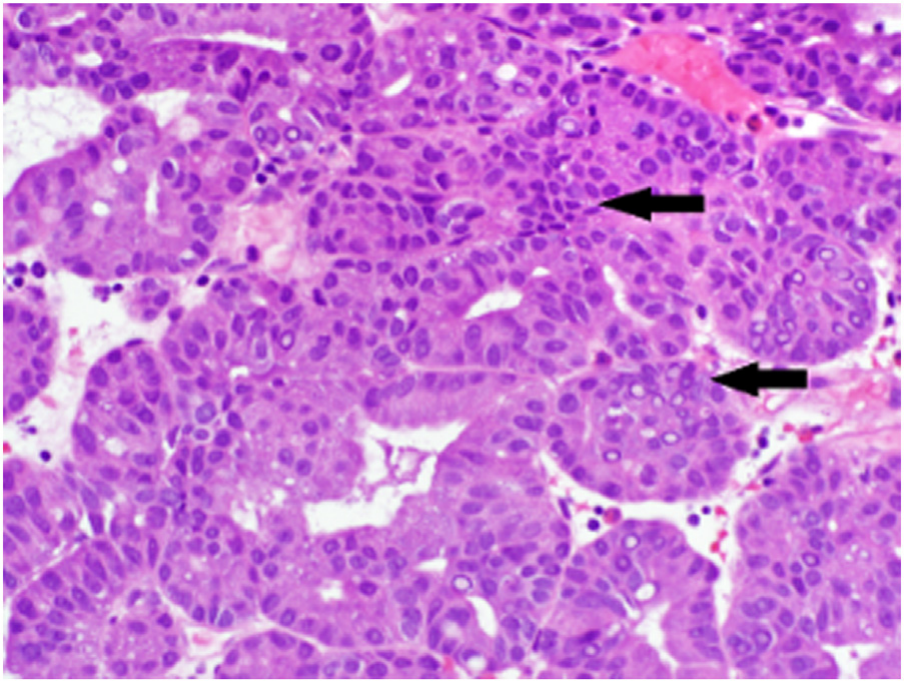
Focal high-grade dysplasia, without apparent invasion (H&E, orig. mag. x100).

We highlight the case of an 86-year-old man who was evaluated for persistent epigastric pain. Notable medical history includes choledocholithiasis requiring multiple previous ERCPs. The patient underwent MRCP, which demonstrated multiple filling defects in the biliary system, including a large defect near the hepatic confluence (Fig. 1). Our patient underwent ERCP during which a large filling defect at the level of the common hepatic duct was confirmed on cholangiogram. Balloon extraction yielded polypoid material rather than stones as expected (Fig. 2). Histology revealed intraductal tubulopapillary neoplasm of the bile duct with predominant low-grade dysplasia and focal high-grade dysplasia (Fig. 3, *arrow* indicating area of high-grade dysplasia). There was no evidence of invasive adenocarcinoma.

**Figure 4:**
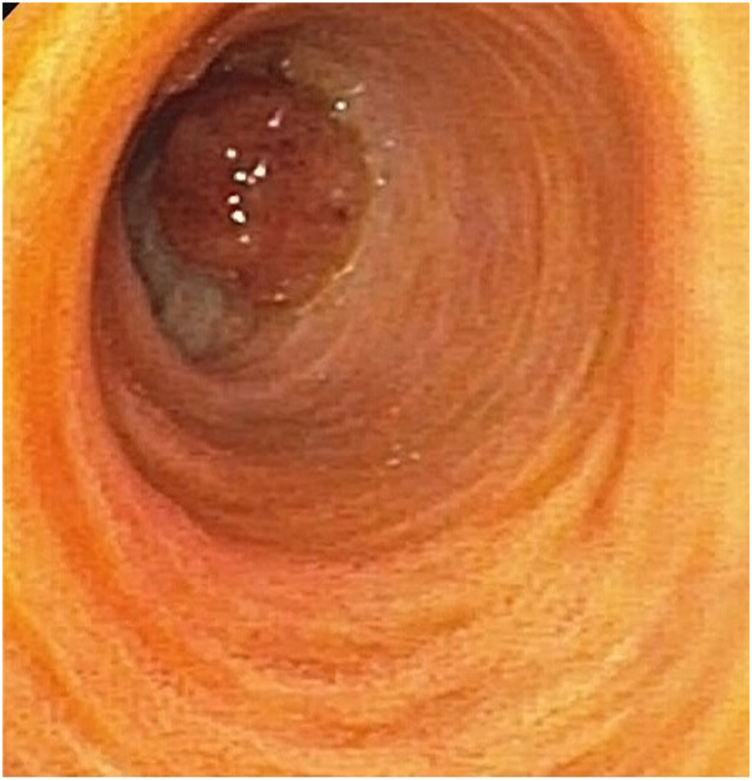
Endoscopic view of the biliary polypoid lesion using direct peroral cholangioscopy.

After multidisciplinary discussion involving the patient and noting his age and comorbidities, endoscopic resection using DPOC was undertaken. First, biliary cannulation was readily achieved through the prior sphincterotomy over a 0.035-inch angled Glidewire (Olympus, Center Valley, Pa), and sphincteroplasty was undertaken with a through-the-scope balloon dilator to a maximum of 12 mm (Video 1, available online at www.giejournal.org). The duodenoscope was then withdrawn and a pediatric gastroscope was used to intubate the dilated biliary orifice and advanced to the bifurcation via an array of maneuvers, including enteroscope stiffening wire, patient repositioning, and external abdominal pressure. The polypoid lesion was visualized endoscopically and measured approximately 1.2 cm (Fig. 4). The intraductal polyp was grasped in its entirety using a pediatric snare (pediatric polypectomy snare, 2.5-cm loop; TeleMed, Hudson, Mass) and resected using ConMed electrocautery (18W coagulation current). The initial attempt to treat the base of the resected polyp with argon plasma coagulation (Beamer argon probe, 1.8 mm × 160 cm; ConMed, Utica, NY) was limited by suboptimal visualization. Therefore, the decision was made to switch to biliary radiofrequency ablation (RFA). The duodenoscope was reinserted and an 8F biliary RFA catheter (Habib EndoHPB; Boston Scientific, Marlborough, Mass) was used to deliver thermal therapy to the area of the common hepatic duct and confluence over a period of 90 seconds, with good treatment effect.^3^ Given the very dilated caliber of the bile duct, a stent was not placed. Subsequent ERCP 3 months after the initial endoscopic resection demonstrated a focal stricture at the level of the common hepatic duct. The stricture was evaluated endoscopically using dedicated direct cholangioscopy (SpyGlass; Boston Scientific) and appeared inflammatory and likely secondary to recent RFA therapy (Video 1). This instrument was used over DPOC given prior challenges in performing DPOC proximally to the common hepatic duct at the time of the biliary polyp resection. There was no evidence of lesion recurrence at the prior resection site. The stricture was treated by placement of a transpapillary 10-mm × 10-cm fully covered self-expendable metal stent (Viabil; Gore Medical, Flagstaff, Ariz) across the stricture. Follow-up ERCP for stent removal and DPOC 18 months and then at 30 months after index resection demonstrated no neoplastic or stricture recurrence. The patient remains well to date with normal liver tests and no symptomatic recurrence of pain or cholangitis.

## Discussion

**Figure 5:**
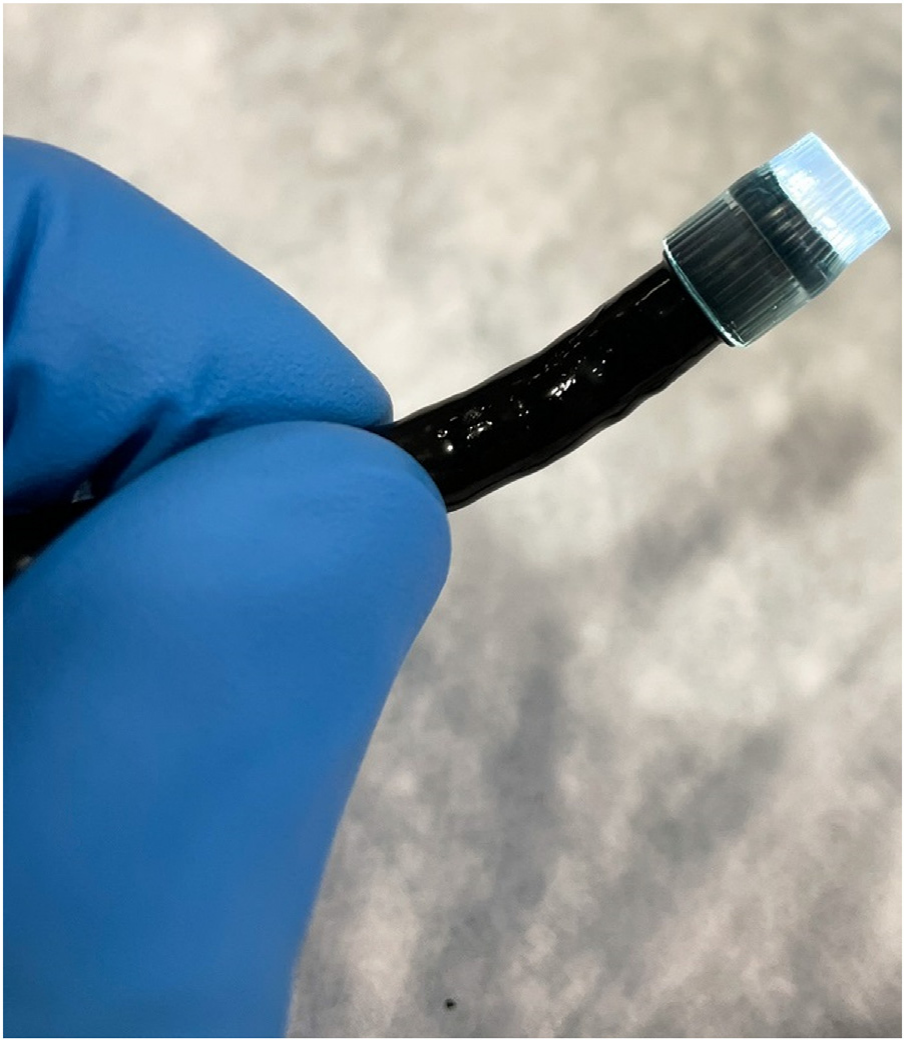
Custom-made distal cap from suction tube.

**Figure 6:**
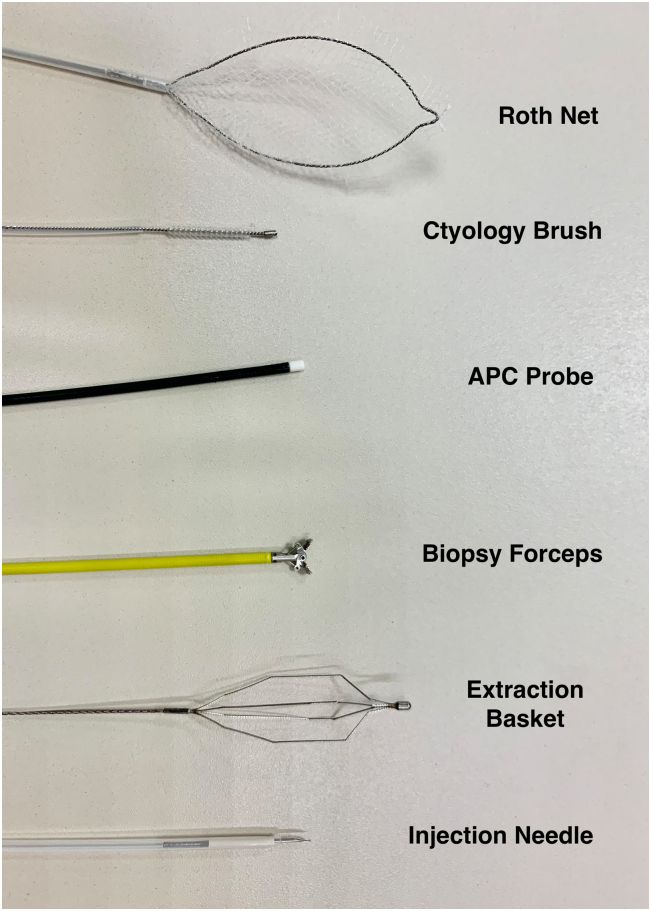
Commercially available equipment to perform interventional direct peroral cholangioscopy through pediatric gastroscope.

The indications for the use of the ultra-slim pediatric endoscope has broadened significantly. Its ultra-thin caliber allows transnasal endoscopy to be performed, as well as passage through the small-caliber GI lumen.^4^ Our case demonstrates an effective endoscopic resection of a biliary polypoid neoplastic lesion using DPOC with a pediatric gastroscope and several accessories followed by biliary RFA to consolidate treatment effect and reduce disease recurrence. This important yet seldom performed advanced endoscopic technique has been previously described for the management of intrabiliary polypoid or mass lesions.^5^^,^^6^ It is important to emphasize that prior sphincterotomy and often balloon sphincteroplasty are prerequisites to permit direct biliary intubation with the pediatric gastroscope. Conventional intubation techniques involve advancing the pediatric gastroscope to the second portion of the duodenum, past the major papilla. Retroflexion is then performed in the second portion of the duodenum with the pediatric gastroscope in a long or semi-long position. The scope is then gently withdrawn while applying counterclockwise torque to reduce the loop (Video 1). This maneuver usually results in deep biliary intubation. While we were able to achieve deep biliary cannulation using a free-hand technique with the aforementioned maneuvers and additional abdominal pressure, the presence of a guidewire may also help facilitate deeper access.^1^ Furthermore, a pediatric occlusion balloon catheter can be used and advanced over the guidewire into the bile duct. The anchoring balloon is then inflated to provide additional stability during biliary intubation.^7^ A custom-made clear cap (MEDI-VAC tubing, 5 mm; Cardinal Health, Springfield, Ill) can be attached to the pediatric gastroscope tip to aid in initial biliary intubation and may offer increased stability while performing interventional procedures such as intraductal snare resection (Fig. 5). The 2-mm accessory channel of the pediatric gastroscope allows easier passage of numerous commercially available devices permitting biliary interventions (Fig. 6). Possible interventions include snare resection, argon plasma coagulation, endoscopic-directed biopsies of the biliary mucosa, foreign body retrieval, laser, and electrohydraulic lithotripsy. Air insufflation within the biliary tree is actively discouraged as this has been associated with air embolization leading to severe adverse implications.^7^ We used carbon dioxide (CO_2_) insufflation to allow better visualization of the biliary mucosa and the polypoid lesion. Caution to avoid excessive CO_2_ insufflation of the biliary tree is advised, and while not used in this case because of the large biliary orifice and ducts, performing DPOC with a concomitant “venting” stent may mitigate embolization or barotrauma risk particularly if performing intraductal argon plasma coagulation. Sterile saline irrigation through the accessory channel of the ultra-slim endoscope without CO_2_ insufflation has been described and is an effective technique in achieving direct visualization of the biliary mucosa while mitigating the risks of air embolization.^7^ As shown in this case, biliary stricture may occur even in very large bile ducts after RFA; therefore, prophylactic stent placement should be considered in all cases.

In summary, DPOC is an important but seldom performed endoscopic procedure given the associated technical challenges. It offers both diagnostic and therapeutic capability and is economically favorable compared to a single-use cholangioscopy. We have described technical aspects of performing DPOC and highlighted available equipment.

## Disclosure

Dr Storm reports research support from Apollo Endosurgery, Enterasense, Endo-TAGSS, and 10.13039/100008497Boston Scientific and consulting for Apollo Endosurgery, ERBE Elektromedizin, GI Dynamics, Olympus, and Intuitive. All other authors disclosed no financial relationships.

## Supplementary data

Video 1 Demonstration of techniques for performing direct peroral cholangioscopy.
